# Measles Transmission in an International Airport at a Domestic Terminal Gate — April–May 2014

**Published:** 2015-06-26

**Authors:** Emily Banerjee, Cynthia Hickman, Kathryn Engels, Cynthia Kenyon

**Affiliations:** 1 Minnesota Department of Health

On April 22, 2014, the Minnesota Department of Health notified CDC of a case of measles in a child aged 19 months who had documentation of receiving 1 dose of measles, mumps, and rubella vaccine at age 12 months. The child’s illness was clinically compatible with measles, which was confirmed by polymerase chain reaction and immunoglobulin M serology at the Minnesota Department of Health Public Health Laboratory. The child was febrile and developed a rash on April 17 while on an international flight from India to the United States before taking a connecting flight from Chicago to Minneapolis. Persons with measles are infectious from 4 days before to 4 days after rash onset (1). Therefore, travelers were exposed on both the international and domestic flights. CDC’s Division of Global Migration and Quarantine was contacted and provided information on potentially exposed persons to relevant health departments for follow-up. No documented transmission was reported as a result of the two flight exposures.

On May 5, the Massachusetts Department of Public Health contacted the Minnesota Department of Health to report a case of measles in a Minnesota resident aged 46 years with unknown vaccination status, who was traveling in Massachusetts for business when a rash was observed. The case was confirmed by polymerase chain reaction and immunoglobulin M serology at the Massachusetts Department of Public Health Laboratory. This person had no known exposures or international travel, and did not fly on the same aircraft as the child from Minnesota on April 17. However, investigation revealed that both patients had traveled through a Chicago airport and used the same gate for their respective flights. Measles is a highly communicable disease, and infectious droplets can remain suspended in the air for up to 2 hours after an infected person leaves the area (2).

Although transmission could have occurred anywhere in the airport where the child and the adult shared airspace, it most likely occurred in the gate area during the 46-minute interval between the arrival of the adult’s flight and the scheduled departure of the child’s flight. The airline confirmed that domestic flights board 30–45 minutes before departure, and families with children typically board first. The child’s family likely would have been preparing to board near the front of the gate area when the arriving adult exited his aircraft and passed through the area. Both cases were genotyped as D8 (endemic in India, where the child evidently acquired measles), and the corresponding nucleotide sequences were determined to be identical. The adult was admitted for isolation only at a Massachusetts hospital during the last 5 days of his infectious period. The child was admitted for 3 days at a Minnesota hospital. Both recovered fully without complications.

Measles transmission at international airports has been documented previously (3). Airport settings facilitate the mixing of persons from countries where measles is endemic around the world. The infectiousness of measles is evident when considering that transmission in this case occurred at a domestic terminal during a short period with brief contact.

Vaccinated persons can acquire measles for various reasons, including primary or secondary vaccine failure or improper vaccine storage, handling, or administration; however, measles transmission from a vaccinated person is rare (4). Although primary vaccine failure was not laboratory confirmed in this case, the child’s highly elevated acute immunoglobulin M serology result and classic clinical presentation were consistent with immunologic naïveté before infection (4). This incident also underscores the importance of CDC’s recommendation for international travelers aged ≥12 months to receive 2 doses of measles, mumps, and rubella vaccine separated by at least 28 days, with the first dose administered at age ≥12 months (1).
